# Hypoxia-inducible factor-driven glycolytic adaptations in host-microbe interactions

**DOI:** 10.1007/s00424-024-02953-w

**Published:** 2024-04-04

**Authors:** Emily DeMichele, Andre G. Buret, Cormac T. Taylor

**Affiliations:** 1 School of Medicine and Systems Biology Ireland, The Conway Institute, University College Dublin, Belfield, Dublin 4 Ireland; 2 Department of Biological Sciences, University of Calgary, Calgary, AB Canada

**Keywords:** Hypoxia, Glycolysis, Glucose, Bacteria, Infection

## Abstract

Mammalian cells utilize glucose as a primary carbon source to produce energy for most cellular functions. However, the bioenergetic homeostasis of cells can be perturbed by environmental alterations, such as changes in oxygen levels which can be associated with bacterial infection. Reduction in oxygen availability leads to a state of hypoxia, inducing numerous cellular responses that aim to combat this stress. Importantly, hypoxia strongly augments cellular glycolysis in most cell types to compensate for the loss of aerobic respiration. Understanding how this host cell metabolic adaptation to hypoxia impacts the course of bacterial infection will identify new anti-microbial targets. This review will highlight developments in our understanding of glycolytic substrate channeling and spatiotemporal enzymatic organization in response to hypoxia, shedding light on the integral role of the hypoxia-inducible factor (HIF) during host–pathogen interactions. Furthermore, the ability of intracellular and extracellular bacteria (pathogens and commensals alike) to modulate host cellular glucose metabolism will be discussed.

## Glucose metabolism and the hypoxia-inducible factor (HIF)

### Glucose metabolism

**Fig. 1:**
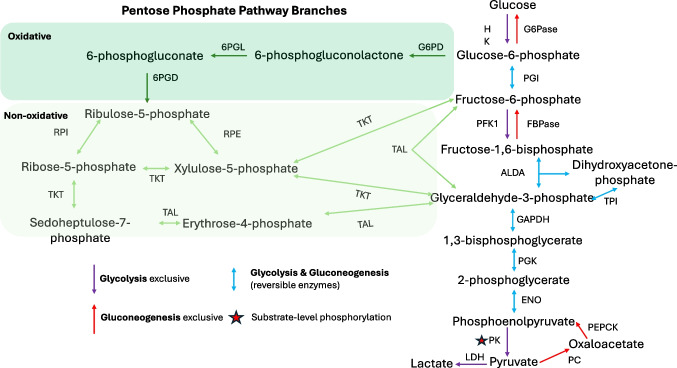
Glycolytic, gluconeogenic, and pentose phosphate pathways. Glycolysis breaks down glucose via the concerted action of hexokinase (HK), phosphoglucose isomerase (PGI) phosphofructokinase 1 (PGK1), aldolase A (ALDA), triosephosphate isomerase (TPI), glyceraldehyde 3-phosphate dehydrogenase (GAPDH), phosphoglycerate kinase (PGK), enolase (ENO), and pyruvate kinase (PK). Pyruvate can then be fermented to lactate via lactate dehydrogenase (LDH) or enter gluconeogenic metabolism via the action of pyruvate carboxylase (PC) to form oxaloacetate, followed by conversion to phosphoenolpyruvate via the action of phosphoenolpyruvate carboxykinase (PEPCK). Gluconeogenesis produces glucose from pyruvate, utilizing many of the same enzymes of glycolysis. However, gluconeogenesis differs from glycolysis with the use of fructose 1,6-bisphosphatase (FBPase) and glucose-6-phosphatase (G6Pase). The pentose phosphate pathway (PPP), illustrated in green boxes/arrows, can interconvert glucose and glycolytic metabolites to produce substrates for nucleic acid synthesis. The oxidative branch (darker green) converts glucose-6-phosphate to ribulose-5-phosphate via the action of glucose 6-phosphate dehydrogenase (G6PD), 6-phosphogluconolactonase (6PGL), and 6-phosphogluconate dehydrogenase (6PGD). The non-oxidative branch uses ribose 5-phosphate isomerase (RPI), ribulose 5-phosphate epimerase (RPE), transketolase (TKT), and transaldolase (TAL) to further interconvert sugars, feeding glyceraldehyde-3-phosphate and fructose-6-phosphate back into glycolytic/gluconeogenic metabolism

Cellular metabolism is a dynamic and orchestrated process, the nature of which is dependent upon environmental conditions, primarily glucose and oxygen availability. Glucose is the main substrate utilized by cells across all kingdoms to generate the common cellular energetic currency, adenosine triphosphate (ATP). Eukaryotic cells facilitate glucose uptake via both passive (glucose transporters (GLUT)) and active (sodium-glucose co-transporters (SGLT)) mechanisms [1]. Following cellular uptake and phosphorylation, glucose is converted to pyruvate through glycolysis. The glycolytic pathway relies on the concerted action of 10 enzymes, the rate-limiting enzyme being phosphofructokinase 1 (PFK1) (Fig. 1) [2]. Glycolysis yields a net of 2 molecules of ATP via substrate-level phosphorylation (SLP). Although this process does not require oxygen, the production of pyruvate is required for the completion of subsequent aerobic metabolism by the tricarboxylic acid cycle (TCA) and electron transport chain (ETC). The TCA cycle and ETC enable the mitochondrial ATPase to produce a further 36 molecules of ATP per molecule of glucose consumed, using oxygen as a terminal electron acceptor, thereby yielding a total of 38 molecules of ATP. However, under anaerobic conditions, glycolysis is followed by fermentation to regenerate NAD+ via the production of lactate by lactate dehydrogenase (LDHA) yielding a net of just 2 ATP per molecule of glucose consumed. Therefore, under conditions where mitochondrial metabolism is inhibited, including during hypoxia, cells must increase the rate of glycolysis by approximately 17-fold to maintain the same levels of ATP as in normoxia [3]. It has recently become appreciated that, as well as facilitating ATP production, increased rates of glycolysis can have important consequences for cell phenotype and function including immune cell activity [4].

While glycolysis acts as a catabolic process for cells, anabolic pathways also use some of these enzymes in a reversible fashion. Gluconeogenesis is the anabolic reciprocal to glycolysis, utilizing the reversible action of glycolytic enzymes including aldolase (ALDA), glyceraldehyde 3-phosphate dehydrogenase (GAPDH), phosphoglycerate mutase (PGM), and enolase (ENO1) to produce glucose from pyruvate (Fig. 1). This metabolic cycle is paramount when cellular glucose stores are depleted, conferring a secondary mechanism by which cells can regulate glucose availability. Glycolysis and gluconeogenesis do however also possess enzymes that are exclusive to each cycle. For example, the last step of glycolysis is catalyzed by the irreversible enzyme pyruvate kinase (PK), while the first step of gluconeogenesis is catalyzed by the irreversible enzyme phosphoenolpyruvate carboxy kinase (PEPCK) [5]. The rate-limiting enzymes of the two processes also differ as glycolysis is controlled by the action of hexokinase 2 (HK2) and phosphofructokinase 1 (PFK1), while gluconeogenesis is controlled by glucose-6-phosphatase (G6Pase) and PEPCK [6, 7]. Differences in rate-limiting enzymes ensure the processes can be reciprocally regulated by different intermediates and energetic substrates, allowing cells to tightly control glucose concentrations.

Another metabolic cycle associated with glycolysis that can be enhanced under conditions of glucose deprivation is the pentose phosphate pathway (PPP). The PPP can utilize pyruvate, as well as other glycolytic intermediates to promote the synthesis of nucleotides via the oxidative branch, making it a key pathway during periods of proliferation (Fig. 1) [8]. Ribulose-5-phosphate is the key product of this process. The oxidative branch also results in the production of nicotinamide adenine dinucleotide phosphate (NADPH), a key reactive oxygen species (ROS) scavenger. In addition, the PPP possesses a non-oxidative branch which has a more prominent role in redox homeostasis as it allows for the interconversion of sugars [8]. The fate of glucose entering the PPP and glycolysis hinges on the action of HK, as well as the bimolecular needs of the cell. Importantly, a reversible link between glycolysis and the PPP also exists. Both transketolase and transaldolase in the PPP can produce glucose-3-phosphate and fructose-6-phosphate, feeding substrates into glycolysis (Fig. 1). Given glucose plays an integral role in the sustained maintenance of multiple metabolic processes, changes in glucose availability will inevitably alter the cells’ metabolic profile and energetic requirements.

Cancer cells capitalize on the glycolytic production of ATP regardless of oxygen availability. Increased glycolytic flux and lactate production are hallmarks of cancer cell metabolism, termed the Warburg effect [9, 10]. While the reason for this metabolic shift remains controversial, it is believed cancer cells repurpose mitochondrial enzymes for the production of macromolecules and rely on glycolysis instead for bioenergetic maintenance. However, cancer is not the only instance in which a Warburg-like metabolic shift can be observed. Immune cells also exhibit the capacity to skew their metabolic flux to favor glycolysis in response to environmental signals. For example, enhanced glycolysis drives cytokine production by dendritic cells, infiltrating the capacity of both M1 macrophages and dendritic cells, as well as differentiation and activation of T cells [4]. Furthermore, sites of inflammation where immune cells are recruited are often devoid of oxygen, augmenting this immuno-metabolic shift [11]. The molecular mechanisms underpinning adaptive and innate immune cell metabolism and responses to tissue hypoxia are therefore inherently linked. Indeed, numerous infection and disease states are characterized by increases in cytokine production and inflammatory markers that drive tissue hypoxia and reciprocally may be produced in response to oxygen depletion [12, 13]. This highlights the importance of glucose metabolism in host responses to both intra- and extracellular insults, such as those inflicted by bacterial pathogens.

### Regulation of glucose metabolism by HIF

Given the importance of glycolysis in maintaining ATP homeostasis and controlling cell fate, it is paramount to understand the mechanisms regulating the rate of glycolysis in cells. One of the most influential regulators of mammalian cellular metabolism is the hypoxia-inducible factor (HIF). This constitutively produced transcription factor activates hundreds of genes that aid in cellular survival when oxygen levels subvert the threshold required to maintain aerobic metabolism. HIF is a heterodimer, consisting of an oxygen-dependent cytoplasmic alpha subunit, of which three isoforms have been identified, and a nuclear beta subunit [14–16]. HIF is regulated tightly by the presence of oxygen in the cytoplasm of cells. Under normoxic conditions, hydroxylases, including prolyl hydroxylases (PHDs) and factor inhibiting HIF (FIH), utilize oxygen as a substrate to tag conserved proline or asparagine residues on HIFα, respectively [17–19]. These hydroxyl tags elicit recognition by the von Hippel-Lindau protein, leading to subsequent ubiquitination and 26S proteasomal degradation of HIFα [20, 21]. Hence, under sufficiently oxygenized conditions, the HIFα subunit is ubiquitously and constitutively produced at a high rate but degraded immediately through this oxygen-dependent pathway.

Under hypoxic conditions, PHDs and FIH no longer have sufficient access to oxygen to hydroxylate HIFα, allowing the subunit to stably translocate to the nucleus where it dimerizes with the HIF-1β subunit and cofactors p300 and the CREB-binding protein (CBP) [22]. This complex then binds to hypoxia response elements (HRE) found in the promoter of genes which enable cellular adaptation and survival during hypoxic stress [23]. Notably, the HIF-1α isoform is the key transcriptional regulator of adaptive glucose metabolism under hypoxic conditions, promoting transcription of all ten glycolytic enzymes, as well as GLUT transporters to enhance glycolytic flux during hypoxia [4]. This adaptation is paramount to cells enduring hypoxic conditions as the lack of oxygen availability inhibits aerobic metabolism. Increased glycolytic flux mediated by HIF aids in cellular maintenance of bioenergetic homeostasis in the absence of oxidative phosphorylation. HIF is key to the induction of the Warburg effect in cancer cells as the tumor environment is often characterized by hypoxia due to the cellular proliferative capacity outpacing tumor vascularization [24]. Additionally, HIF-1α plays a key role in the adaptive metabolic shift to favor glycolysis in immune cells as stabilization of the transcription factor is integral to the function of M1 macrophages, T cells, neutrophils, and dendritic cells [11, 25].

### Glycolytic metabolons

A second mechanism by which flux through the glycolytic pathway may be increased involves the formation of glycolytic metabolons. While it has been widely accepted for decades that the cytoplasm is diffuse with substrates and enzymes, recent findings have highlighted the ability of cells to streamline metabolic processes via substrate channeling. To do so, cells can employ a variety of tactics including covalent bonding of enzymatic complexes, formation of intramolecular tunnels, and spatial organization [26]. Given glucose metabolism is integral to cellular survival and relies on the concerted action of many enzymes, it is not surprising that the coalescence of glycolytic and gluconeogenic enzymes has been identified. Glycolytic metabolons have been observed in numerous organisms. For example, protozoa including *Trypanosoma* sp. possess clusters of glycolytic and gluconeogenic enzymes which form membrane-bound organelles called glycosomes [27]. In *Drosophila*, interactions between glycolytic enzymes including PGM, ALDA, and GAPDH are localized to flight muscle tissue, mediated in part by interactions with sarcomeres and the TRIM32 protein [28, 29]. In *C. elegans*, glycolytic enzymes cluster with scaffolding proteins in presynaptic neurons during energetic stress due to hypoxic conditions [30]. Similarly, yeast can form granules termed G bodies that accelerate glucose consumption and metabolism during hypoxia [31, 32]. However, many questions remain with respect to the structure and function of such glycolytic complexes.

In mammalian cells, the clustering of glycolytic enzymes has also been documented. First visualized in breast, cervical, and pancreatic cancer cell lines, liver-type phosphofructokinase I (PFKL) tagged with a monomeric enhanced green fluorescent protein (mEGFP) colocalizes with pyruvate kinase M2 (PKM2) and PEPCK with ~ 85% efficiency [33]. These dynamic structures were first termed glucosomes (syn. glycoplex, hereafter glycolytic metabolon). Interestingly, glycolytic metabolons also contain gluconeogenic enzymes. Fructose 1,6-bisphosphatase (FBP), a gluconeogenic enzyme that catalyzes the reverse reaction of PFKL, was consistently localized in these complexes [33]. This finding suggests the reciprocal regulation of these enzymes may be a result of their direct interaction [33].

Glycolytic metabolon size also has a pivotal role in cellular metabolic activity and fate. Three sizes of glycolytic metabolons were identified by Kohnhorst et al. in human breast carcinoma cells (Hs578T): small being less than 0.1 μm, medium being 0.1–3 μm, and large being 3–8 μm. The size of the enzymatic complex is correlated directly with the cellular metabolic profile and has implications for the cell’s progression through the cell cycle. Medium-sized glycolytic metabolons are more abundant when the PPP is accelerated using methylene blue [33]. Also, the diversion of glucose to serine biosynthesis via treatment with epidermal growth factor (EGF) favors the assembly of large glycolytic metabolons due to temporal activation of ERK1/2 [33]. This activation is a result of increased ERK phosphorylation during EGF treatment, corroborated by the decrease in glycolytic metabolon size in Hs578T cells after treatment with an ERK inhibitor or in cells with ERK1/2 knockdowns [34]. Relative protein abundance of glycolytic enzymes previously identified to localize in these clusters is not altered upon inhibition of ERK1/2, indicating the SRE family kinases modulate the formation of these structures on an organizational level rather than altering enzyme expression [34]. Intriguingly, when cells are treated with inhibitors of the G1/S or G2/M checkpoints, the abundance of small-sized glycolytic metabolons increases drastically [35]. A recent study has also illustrated the prominent role of lactate in cell cycle regulation. Increased production of this fermentation product as a result of increased glycolytic flux can directly inhibit the action of SUMO protease sentrin isopeptidase 1 (SENP1), stabilizing SUMO tags on the anaphase-promoting complex (APC/C) in human cells which subsequently promotes mitotic exit [36]. Taken together, these findings illustrate the influence of cellular metabolism on the cell proliferative state, highlighting the close association between glycolytic metabolons and cell cycle regulation.

Delving deeper into the mechanism underlying the formation of glycolytic metabolons, qualitative high-content high-throughput screening assays were carried out on PFK1-mEGFP HeLa cells to identify which pharmacological inhibitors could influence metabolon formation [37]. Three main kinase targets, CDK2, RSK, and AURKA, were determined to have a profound impact on glycolytic metabolon formation [37]. Reduction in expression of all three kinases individually with shRNAs decreases the abundance of small glycolytic metabolons, indicating cells likely shunt glucose to the PPP or serine biosynthesis when the cell cycle is slowed or dysregulated [37]. This finding further ameliorates the favoring of glycolytic metabolism during proliferative states.

### Role of HIF in glycolytic metabolons

While HIF can directly upregulate the expression of all glycolytic enzymes and glucose transporters (i.e., GLUT1, GLUT4), the transcription factor can employ other mechanisms to alter cellular metabolism during hypoxia. Post-translational modifications including SUMOylation have proven to indirectly accelerate HIF-1α stabilization, resulting in increased glycolytic enzymatic activity [38, 39]. During hypoxia, the PI3K (phosphatidylinositol-4,5-bisphopshate 3 kinase) Akt (protein kinase B) pathway acts to accelerate HIF stabilization and glycolytic metabolism while inhibiting glycogen synthesis [40]. Furthermore, this pathway results in the production of platelet-derived growth factor which subsequently reduces the activity of mitochondrial complex IV [41, 42]. Also, HIF plays a role in reduced cellular oxygen metabolism by targeting mitochondrial genes, largely through the action of PDK1 [43]. PDK1, in coordination with LDHA, shunts pyruvate away from the TCA cycle and inactivates the pyruvate dehydrogenase complex [43]. Overall, HIF employs a multifaceted approach to altering cellular metabolism.

Beyond the ability of HIF to modulate glucose metabolism directly or indirectly at the transcriptional level, recent findings have suggested that HIF-1α has a novel role in glycolytic metabolon assembly. Firstly, the treatment of cells with resveratrol, a known inhibitor of HIF-1α, skews glycolytic metabolon assembly to favor large complexes, shunting glucose away from glycolytic metabolism [37, 44]. Secondly, Caco-2 intestinal epithelial cells with a knock down of HIF-1α are incapable of inducing glycolysis in the presence of a hypoxia mimetic [45]. However, Caco-2 cells expressing transcriptionally inactive HIF-1α due to sgRNA interference in the basic helix-loop-helix domain exhibit persistent glycolytic acceleration [45]. Mass spectrometry analysis also indicates the HIF-1α subunit interacts with GLUT1 and PFKP in a hypoxia-dependent manner, suggesting an additional non-transcriptional moonlighting role for HIF-1α in the control of glucose metabolism [45]. Previously, it was thought that the sole function of the stabilized HIF-1α subunit was to translocate to the nucleus to bind the HIF-1β subunit, assemble with cofactors and bind to the hypoxia response element found in hundreds of genes which aid in cellular survival during hypoxia. However, this identification of a non-transcriptional role in which HIF-1α remains localized to the cytoplasm and interacts with glycolytic proteins highlights the versatility in mechanisms by which this protein can influence cellular survival. The interaction of HIF-1α with glycolytic enzymes as well as GLUT1 further suggests the possibility of substrate channeling given the glycolytic metabolons are localized with a transmembrane protein. Future work is required to further understand the role of HIF-1α in modulating the functional capacity and size of glycolytic metabolons. In summary, increased glycolytic flux is part of an important adaptive bioenergetic response to hypoxia which has important implications for host–pathogen interactions due to its effects on cellular immune function.

## Host-microbe interactions and glycolytic metabolism

### Bacterial pathogens and host metabolism

During bacterial infections, cells must employ defense strategies against microbes to combat pathogenicity. However, bacteria possess a diverse array of virulence factors and exhibit tissue specificity to combat host responses. Furthermore, bacteria can function as intracellular or extracellular pathogens. Both modes of bacterial infection have proven capable of modulating host metabolic responses in immune and epithelial cells. Glucose is also a common carbon source utilized preferentially by many bacteria, and hence, bacteria can compete with the host for this energetic substrate. While the direct consumption of host carbohydrates by bacteria can alter host metabolic flux, bacteria may also alter HIF activation in the host [46]. Firstly, aerobic microbes can render the infected tissue hypoxic due to increased localized oxygen consumption [47, 48]. Secondly, many bacterial species secrete siderophores to sequester iron. Given iron is a key cofactor for PHD activity, bacteria can indirectly activate HIF via the depletion of host iron pools [47]. Thirdly, the recognition of bacterial membrane proteins including lipopolysaccharides (LPS) by immune cells can lead to HIF stabilization due to the activation of nuclear factor kappa B (NFκB) [49]. In all, there are multiple mechanisms by which bacteria can directly or indirectly modulate host cell metabolism. This section will explore what is known regarding host glycolytic alterations induced by intra- and extracellular bacteria.

### Intracellular bacteria

**Fig. 2:**
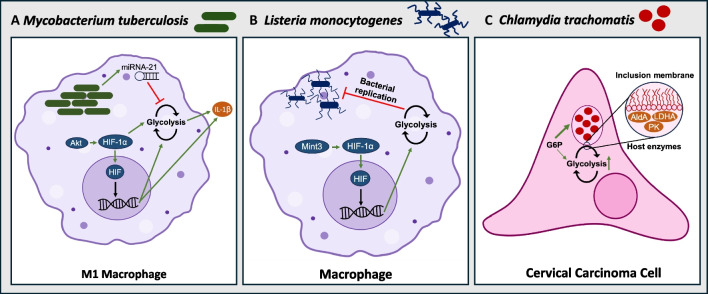
Glycolytic metabolism modulation by intracellular bacterial pathogens. **A** Modulation of M1 macrophage glycolytic metabolism in *Mycobacterium tuberculosis* (Mtb) infection. Mtb upregulates miRNA-21 in host macrophages, inhibiting glycolysis. However, the host upregulates Akt/HIF-1α signaling to increase HIF-driven glycolysis, leading to increased production of IL-1β. **B** Modulation of macrophage glycolytic metabolism in *Listeria monocytogenes* infection. In response to *L. monocytogenes*, host macrophages activate HIF-1α via Mint3, leading to enhanced glycolysis which subsequently reduces bacterial replication. **C** Modulation of cervical carcinoma cell metabolism in *Chlamydia trachomatis* infection. Host glycolytic enzymes (aldolase A (ALDA), lactate dehydrogenase A (LDHA), and pyruvate kinase (PK)) are localized to the inclusion membrane of the bacterium, and glucose-6-phosphate (G6P) is preferentially shuttled into the inclusions

Obligate intracellular microbes must compete for cytoplasmic substrates in the host cell. Hence, multiple studies have examined the ability of these pathogens to alter host metabolism. A Warburg-like shift has been characterized in numerous cell types as a result of infection with intracellular bacteria. It is important to note that many studies utilize cancer cells which exhibit a basal reliance on glycolysis that exceeds non-cancerous cell levels. The use of primary cells is therefore an important consideration for infection models that assess metabolic shifts. Of interest, recent evidence indicates that commensal gut bacteria may become invasive intracellular pathobionts during enteric infections, highlighting the role of transkingdom interactions in bacterial pathogenicity [50–53]. More research is warranted to assess the effects of pathobiont invasion on epithelial hypoxia. In this section, we will highlight what is known regarding the modulation of host metabolism in the context of common intracellular pathogens including *Mycobacterium tuberculosis*, *Listeria monocytogenes*, and *Chlamydia trachomatis* (Fig. 2).

*Mycobacterium tuberculosis* (Mtb) is the causative agent of tuberculosis, the second top pathogenic killer in the world in 2022 [54]. This intracellular pathogen can remain latent in a host as only an estimated 5–10% of individuals infected with Mtb develop an infection during their lifetime, most commonly affecting the pulmonary system [55]. While Mtb can infect numerous cell types, modulation of host metabolism by Mtb has been best studied in macrophages. Macrophages can be activated into an M1 or M2 phenotype; the M1 phenotype exhibits a predominantly glycolytic metabolic profile while M2 macrophages rely on OXPHOS for ATP generation [56]. M1 macrophages are better suited for combatting Mtb infections as their Warburg-like metabolism, driven by an increase in IL-1β and subsequent HIF-1α activation, reduces intracellular load and survival of Mtb [57–60]. This innate pro-inflammatory response is driven by kinases that regulate HIF-1α activity, as knock down of Akt in RAW 264.7 murine macrophages results in a decrease of intracellular lactate accumulation, concomitant with increased Mtb bacterial load [61]. However, the regulation of glycolysis has proven to be multifactorial, as the use of histone deacetylase inhibitors in human monocyte-derived macrophages accelerates macrophage glycolysis and production of Il-1β upon Mtb infection [57]. To combat the host metabolic response, Mtb can also employ transcriptional mechanisms to promote an anti-inflammatory phenotype and repress glycolysis. Micro-RNAs (miRNA) are known to regulate macrophage function, and miRNA-21 in particular is significantly upregulated by Mtb infection [59]. Importantly, miRNA-21 downregulates the production of IL-1β while also targeting the phosphofructokinase muscle isoform (PFK-M), mitigating the anti-mycobacterial response of macrophages [59]. The upregulation of miRNA-21 is a key virulence strategy of Mtb which attenuates immunometabolic responses to infection in macrophages, highlighting the importance of the glycolytic switch in immune cell defense against pathogens [59]. Given Mtb aims to dampen host glycolysis during infection, augmentation of these responses may be key in promoting infection clearance and must be further investigated moving forward.

*Listeria monocytogenes* is a facultative intracellular pathogen capable of replicating in the host cell cytoplasm [62, 63]. This gram-positive microbe causes listeriosis as it can travel through the blood stream to various organs including the brain and gastrointestinal tract [63]. To assess the ability of the pathogen to alter cellular metabolism and isolate the augmentation of glycolysis by cancer cells, metabolic C^13^ isotope labeling was carried out in two cell lines: mouse bone marrow-derived macrophages (BMDM) and macrophage-like J774A.1 cancer cells. While *L. monocytogenes* drastically enhances the glycolytic metabolism in BMDMs, J774A.1 cells exhibit minimal glycolytic alternations upon infection [64]. Importantly, the basal level of glycolysis occurring in uninfected J774A.1 cells far surpasses the basal glycolytic level in BMDMs [64]. This suggests that the acceleration of glycolytic metabolism is favorable for intracellular replication of the pathogen. Another study investigating the mechanism underlying upregulated host glycolytic capacity in *L. monocytogenes*-infected macrophages identified the X11 family member Mint3 as a key regulator of this response. Mint3 suppresses FIH, indirectly increasing HIF stabilization leading to subsequent glycolytic acceleration on the transcriptional level [65]. Interestingly, Mint3 − / − mice exhibit reduced bacterial burden and increased AIM2/NLRP3 inflammasome activity [65]. Interestingly, macrophages isolated from these knockout mice exhibit enhanced pyroptosis and inflammasome activation, replicating the phenotype observed in wild-type mouse macrophages treated with glycolytic inhibitors (i.e., 2-deoxyglucose (2DG)) [65]. However, another study indicated the acceleration of glycolysis as a result of HIF-1α activation in *L. monocytogenes*-infected macrophages was necessary for pro-inflammatory macrophage differentiation as 2DG treatment dampened pro-inflammatory response and impaired M1 differentiation in both mouse and human macrophages [66, 67]. The liver of *L. monocytogenes*-infected mice treated with 2DG also exhibits a greater bacterial load and decreased secretory activity of CD4 + and CD8 + T cells, suggesting this HIF-mediated glycolytic acceleration also influences T cell differentiation [66]. The opposing observations in pro-inflammatory responses to *L. monocytogenes* linked to glycolytic activity may reflect the differences in the infection due to its systemic nature, and therefore, future studies may benefit from isolating various immune cell populations within an infected organism to determine if these alterations are tissue specific.

*Chlamydia trachomatis* is an obligate intracellular bacterium responsible for one of the most common sexually transmitted diseases in the world, chlamydia [68]. With a niche in the urogenital tract, this bacterium relies on mucosal epithelial cells for energy, as well as a carbon source. While the bacterium does possess most glycolytic enzymes, it lacks HK [69]. Host glucose-6-phosphate is therefore required for the bacterium to produce ATP, modulating host glycolytic flux quickly upon infection [70]. In HeLa cells, the clustering of glycolytic enzymes to the inclusion membrane of the intracellular bacterium has been visualized [71]. These inclusions reliably included host ALDA, PK, and LDHA, suggesting host cells employ a strategy to enhance glycolytic flux to account for the metabolic burden incurred by *C. trachomatis* [71]. ALDA is the most influential enzyme in the assembly of these clusters, as treatment of HeLa with siRNA against the enzyme notably decreases the size of the inclusions and depletes the production of infectious progeny. Furthermore, glucose-6-phosphate isomerase and 6-phosphofructokinase are upregulated upon infection [71]. Pyruvate dehydrogenase kinase (PDK), the enzyme responsible for the phosphorylation and subsequent inhibition of the pyruvate dehydrogenase complex, was also identified as a key enzyme in infectious progeny replication [70]. Another study evaluating host metabolic changes in immortalized human endocervix cells found no change to cellular ATP levels or OXPHOS upon infection [72]. However, there is an increase in the extracellular acidification rate after infection with *C. trachomatis*, and cells subsequently treated with an LDHA/B inhibitor (GNE-140) illustrate a dose-dependent reduction in infectious progeny [72]. Taken together, these findings stress the importance of glycolysis in the infectious capacity of *C. trachomatis* and highlight the prominent glycolytic shift induced in host cells to sustain bacterial replication.

### Extracellular bacteria

**Fig. 3:**
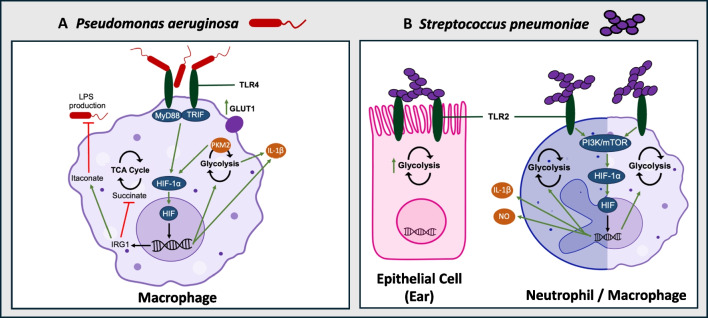
Glycolytic metabolism modulation by extracellular bacterial pathogens. **A** Modulation of macrophage glycolytic metabolism in *Pseudomonas aeruginosa* infection. *P. aeruginosa* interacts with TLR4, activating the MyD88 and TRIF signaling cascades, leading to HIF-1α activation. This in turn increases glycolytic flux, while driving the production of IL-1β. Host cells also upregulate immune response gene 1 (IRG1) to reduce the production of succinate and instead produce itaconate which lowers lipopolysaccharide (LPS) production by *P. aeruginosa*. **B** Modulation of epithelial ear cell metabolism by *Streptococcus pneumoniae* via interaction with TLR2, driving glycolysis. A similar phenomenon is observed in immune cells (neutrophil on the left, macrophage on the right) wherein TLR2 activation drives PI3K/mTOR signaling leading to HIF activation, augmenting host glycolysis. In neutrophils, nitric oxide (NO) and IL-1β production is also attributed to the activation of HIF

While intracellular bacteria can directly modulate or hijack cellular metabolism, extracellular pathogens rely on antigens and pathogen-associated molecular patterns (PAMPS) to alter host cell behavior. For example, LPS, a characteristic PAMP localized to the outer membrane of gram-negative bacteria, can lead to metabolic reprogramming of immune cells including macrophages via activation of the toll-like receptor 4 (TLR4) [73]. Importantly, increased glucose uptake via the active transporter SGLT1 reduces LPS-induced apoptosis in colonic epithelial cells, illustrating the protective role of glucose metabolism in enteric infections [74, 75]. Furthermore, other extracellular microbes including *Campylobacter jejuni* can disrupt TLR9 signaling in enterocytes, reducing intestinal epithelial barrier integrity during infection [76]. This section will highlight what is known regarding the modulation of host metabolism in the context of the common extracellular pathogens *Pseudomonas aeruginosa* and *Streptococcus pneumoniae* (Fig. 3).

*Pseudomonas aeruginosa* is an opportunistic pathogen which can cause pneumonia and poses a lethal risk to cystic fibrosis patients [77]. As a gram-negative microbe, *P. aeruginosa* possesses an outer membrane studded with LPS [78]. Recognition of LPS by TLR4 on the surface of myeloid cells activates numerous signaling cascades via the recruitment of MyD88 and Toll/IL-1R domain-containing adaptor-inducing IFN-β (TRIF) [79]. This subsequently leads to a shift in the metabolic profile of activated pro-inflammatory macrophages, driven by increased GLUT1 glucose uptake and increased activity of glycolytic enzymes (i.e., HK, glucose-6-phosphate dehydrogenase) [80, 81]. The metabolic reprogramming of activated macrophages by TLR4 also heavily employs the action of HIF-1α, as PKM2 expression induced by LPS recognition drives HIF-1α activation and IL-1β secretion [82]. However, the relationship between host immunometabolism and *P. aeruginosa* is more complex than the modulation of glycolysis via TLR4 activation. Increased HIF-1α-driven glycolytic activity in macrophages leads to increased production of succinate, a TCA cycle intermediate which positively regulates glycolysis via inhibition of PHD activity upon accumulation in the cytosol [60, 83]. Succinate is also a preferred carbon source of *P. aeruginosa*, and hence, the increased glycolytic flux aimed to enhance the innate immune response can simultaneously accelerate the proliferative capacity of the bacteria during infection [84, 85]. To lessen the production of succinate, myeloid cells can then activate immune response-gene 1 (*Irg1*), leading to the production of the anti-inflammatory metabolite itaconate [86]. Itaconate combats the oxidative stress induced by enhanced succinate production and dampens glycolytic flux through modification of cysteine residues in ALDA, GAPDH, and LDHA [87, 88]. However, more virulent strains of *P. aeruginosa* are selected in this metabolic environment as itaconate dampens bacterial LPS production while enhancing the production of bacterial extracellular polysaccharides (EPS), enhancing biofilm formation [89]. Overall, there exists a complex bidirectional regulation between host immunometabolism and *P. aeruginosa* expression of LPS versus EPS genes wherein carbohydrate metabolism can dictate bacterial pathogenicity.

Lastly, numerous studies have demonstrated the capacity of *P. aeruginosa* to alter HIF activity. One study determined that HIF-1α expression in colonic epithelial cells enhances the pathogenicity of *P. aeruginosa* by increasing the microbes’ production of potent barrier-dysregulating protein PA-I lectin/adhesin [90]. Opposing this phenomenon wherein HIF-1α benefits the pathogen, another study demonstrated *P. aeruginosa* can reduce HIF-1α expression via the secretion of 2-alkyl-4-quinolone quorum sensing molecules which target HIF for degradation in a PHD-independent manner, reducing expression of HIF-target gene HK [91]. Others have determined epithelial hypoxia reduces internalization of the microbe, and HIF-1α activation is paramount to clearance of ocular *P. aeruginosa* keratitis infections in mice as in leads to increased production of antimicrobials and reduced bacterial load [92, 93]. Further investigation is warranted to fully understand the role of HIF-1α in *P. aeruginosa* infection and its role in modulating host glucose metabolism upon infection.

*Streptococcus pneumonia* is the bacteria responsible for pneumococcal diseases which are localized to the lungs, ears, and nose [94]. The gram-positive microbe primarily uses host glycans found in the mucosa as a source of energy as its metabolism is strictly fermentative [95, 96]. Although it can sustain itself on glycosylated mucins, *S. pneumonia* can alter the metabolic profile of the host depending on the location of the infection. In the lungs of mice infected with *S. pneumonia*, significant decreases in both glycolytic and TCA metabolites have been observed via metabolomic analysis [97, 98]. The mechanism underlying this metabolic attenuation is unknown, but it has been suggested that the catabolism of glucose may be reduced in the lungs during infection to limit substrate production for bacterial replication [99]. Another important consideration is that gram-positive microbes do not possess LPS and hence cannot activate TLR4. Alternatively, gram-positive microbes possess a single membrane of peptidoglycan with lipoteichoic acids, both of which can be recognized by TLR2 [100]. Activation of TLR2 subsequently enhances HIF-1α transcription and activation via the PI3K-mTOR pathway, mimicking the metabolic shift illustrated by TLR4 activation [101]. Hence, recognition of *S. pneumonia* by cells via TLR2 can enhance host cellular glycolytic flux. This metabolic phenomenon has been observed in a mouse model of acute otitis media (AOM), a common ear infection of children often caused by *S. pneumonia* [102, 103]. AOM murine middle ear epithelial cells exhibit upregulated transcription of *hk3*, *pfkfb3*, *pkm2*, *ldha*, and lactate transporter *mct4*, as well as increases in intracellular lactate concentration [102]. Isolated murine neutrophils also required glycolytic activity for effective phagocytosis and killing of *S. pneumoniae* as these abilities were attenuated when neutrophils were treated with 2DG [102]. This effect was attributed to lower HIF-1α activation, suggesting that HIF-1α is the primary regulator of the immunometabolic response to *S. pneumonia* in host neutrophils. HIF-1α has also been linked with macrophage activity in *S. pneumoniae* infection as treatment of macrophages with 2DG similarly lessened the killing of bacteria, a result of lowered NO production and IL-1β production [104]. Taken together, these findings indicate the HIF-glycolytic axis is a key regulator of immune cell responses to *S. pneumonia* infection, but further work is required to understand if TLR2 activation is the primary mechanism by which this microbe drives alternative host glucose metabolism.

### Microbiome dysbiosis and metabolism

The human microbiome is a complex transkingdom community of microbes that play an integral role in bodily homeostasis. While the microbiome consists of bacteria, archaea, fungi, viruses, and protozoa, bacteria are the dominant players existing in a ~ 1:1 ratio with human cells [105]. Although enterotypes have been used in the past to classify an individual’s microbiome based on the dominating bacterial species (Bacteroides, Prevotella, or Ruminococcus), recent computational developments have highlighted the shortcomings of this classification scheme [106, 107]. The composition of the microbiome is dependent on numerous factors including age, geographic location, diet, genetics, and enteric infections. Hence, the microbiome is a dynamic organ that undergoes punctuated shifts throughout an individual’s lifetime, including perturbations that render the microbiome dysbiotic. During dysbiosis, microbes that previously existed as commensals within a biofilm may transition to an invasive pathobiont phenotype, or new pathogenic microbes are introduced that negatively alter host cellular homeostasis [51, 108]. The recent decade has shed light on the role of microbiota dysbiosis in nearly every inflammatory and metabolic condition, highlighting the complex and bidirectional influence of the microbiome on host immunity and metabolism. Furthermore, alterations to the host oxygenic environment resulting in HIF-1α activation can further drive dysbiosis and modulate cellular metabolism.

### Microbiome and influence on host metabolism: SCFAs

While our discussion to date has focused on the role of glycolysis in host–pathogen interactions, most host-microbe interactions are in fact neutral or beneficial to the host. For example, commensal microbes play a central role in human digestion and metabolism. Although humans possess amylases and disaccharidases to break down carbohydrates, we do not possess enzymes to break down complex carbohydrates including starches and plant-derived polysaccharides (cellulose, hemicellulose). Microbes possess a diverse range of carbohydrate-degrading enzymes, allowing them to digest carbs we are unable to [109]. Furthermore, the digestion of complex carbohydrates by microbes fuels the production of short-chain fatty acids (SCFAs). SCFAs, namely acetate, propionate, and butyrate, modulate gut barrier function, host cell metabolism, and immunity, highlighting a prominent indirect way in which microbes impact bodily homeostasis.

Butyrate is a primary energy source for colonocytes, creating a direct link between host and microbial metabolism [110]. Importantly, butyrate oxidation is preferred by colonocytes over glucose oxidation and maximizes cellular oxygen consumption, driving aerobic respiration [111, 112]. This response is mediated by activation of the peroxisome proliferator-activated receptor-γ (PPAR-γ) which subsequently promotes mitochondrial β-oxidation of fatty acids [113, 114]. Butyrate can also inhibit glycolytic activity directly and indirectly via binding to PKM2 and positively regulating PDK expression, respectively [115, 116]. The inhibition of glycolytic flux by butyrate is strong enough to reverse the Warburg shift in colonic cancer cells [115, 117]. Importantly, increased colonocyte oxygen consumption to sustain cellular OXPHOS depletes luminal oxygen concentrations, rendering the colonic lumen anaerobic. A common hallmark of relative bacterial abundance during microbiome dysbiosis is a reduction of butyrate-producing microbes, most of which belong to the phylum Firmicutes [118]. Reduced abundance of butyrate-producing species in the microbiome has been noted in numerous infection and disease states including colorectal cancer, inflammatory bowel disease, irritable bowel syndrome, lupus, diabetes mellitus, obesity, and HIV [119–126]. Some of the key butyrate producers including *Roseburia intestinalis*, *Faecalibacterium prasunitzi*, and *Eubacterium*a*s* are obligate anaerobes and hence do not tolerate elevations in oxygen as a result of altered colonocyte metabolism [127]. This phenomenon constitutes the “oxygen hypothesis,” suggesting that altered colonic metabolism results in the selection of facultative anaerobes, further perpetuating microbiome dysbiosis [128–130]. This cyclical dysregulation highlights the delicate balance between the microbiome and host metabolism.

Not only can butyrate influence the oxygenic environment of the gut, but it is also known to increase mitochondrial respiration though it can reduce ATP turnover via mechanisms that remain incompletely understood [131–133]. Recent evidence suggests the SCFA can promote HIF activation, a possible mechanism by which OXPHOS is inhibited. Butyrate can non-competitively bind to PHDs, acting as a PHD inhibitor [134]. The link between butyrate and HIF activation has also been illustrated in vivo, as mice treated with antibiotics exhibit reduced HIF activation due to butyrate depletion [111]. Subsequent administration of butyrate to antibiotic-treated mice results in increased HIF activation, concomitant with increased tissue oxygenation and barrier integrity [111]. These findings have also been confirmed in the context of *Clostridium difficile* infections, as the protective activation of host HIF-1α is promoted in mice via the administration of exogenous butyrate [135, 136].

### Microbiome and influence on host metabolism: bile salts

Another mechanism by which microbes can modulate host metabolism is via the transformation of bile salts. Bile salts are derivatives of cholesterol synthesized by the liver and aid in digestion, lipid absorption, and toxin elimination [137]. However, the microbial regulation of bile salt composition also has strong implications for glucose metabolism. Bile salts can regulate glucose metabolism through numerous mechanisms. Firstly, they regulate the G-coupled membrane receptor 5 (TGR5) which promotes the release of glucagon-like peptide-1 (GLP-1) which enhances glucose tolerance [138]. Bile salts also regulate the farsenoid X receptor (FXR) receptor which has a key role in glucose homeostasis as FXR-null mice have impaired glucose and insulin tolerance [139, 140]. Modulation of glycolytic enzyme expression (glucose-6-phosphatase) and TCA enzyme expression (fructose-1,6-bisphosphatase) by bile salts has also been documented [141]. Bile salts can repress HIF-1α expression, another potential mechanism by which they alter glycolytic metabolism. Human HepG2 hepatocytes treated with chenodeoxycholate, a primary bile acid, exhibited decreased HIF-1α protein and downregulated expression of HIF-target genes [142]. These observations have also been replicated in lung, breast, prostate, and bronchial epithelial cell lines treated with both chenodeoxycholate and deoxycholate [143, 144]. The mechanism underlying this inhibition and subsequent effect on glycolysis is presently unknown. Overall, bile salts can alter glycolytic metabolism via direct and indirect mechanisms, highlighting another important way in which metabolic functions of the microbiome can alter host glucose metabolism.

## Conclusion

Host metabolism of glucose is a tightly regulated process wherein multiple strategies may be employed dependent on the cells’ metabolic and bioenergetic needs. Under states of oxidative stress, cells enhance glycolytic metabolism to produce energy anaerobically via the action of HIF-1α. Not only can HIF-1α enhance glycolysis at the transcriptional level, but recent studies also suggest it can facilitate the formation of glycolytic metabolons. Importantly, both intra- and extracellular bacterial infections exhibit the capacity to augment cellular glycolytic flux in numerous cell types. However, commensal species can also alter host cellular metabolism suggesting this phenomenon may not be limited to pathogenic conditions. Understanding the mechanism by which cells perturb their metabolism upon interaction with microbes, commensals, and pathogens alike is key for the determination of not only infection pathogenesis but also for basal cellular homeostatic function. Given the broad scope of incidences in which hypoxia can drive inflammation and vice versa, interrogating the role of HIF-1α in host glycolytic shifts will have implications beyond the role of glucose metabolism in host-defense strategies and translate to numerous disease states that are characterized by microbiome dysbiosis. In all, future investigations concerning glycolytic metabolon formation and the role of HIF-1α in the context of host-microbe interactions will be paramount for our understanding of the transkingdom metabolic communications that underpin cellular survival and homeostasis.

## Data Availability

Not applicable.
